# The leaf aqueous extract of *Myrianthus arboreus* P. Beauv. (Cecropiaceae) improved letrozole-induced polycystic ovarian syndrome associated conditions and infertility in female Wistar rats

**DOI:** 10.1186/s12906-020-03070-8

**Published:** 2020-09-11

**Authors:** Marie Alfrede Mvondo, Flavie Ingrid Mzemdem Tsoplfack, Charline Florence Awounfack, Dieudonné Njamen

**Affiliations:** 1 Research Unit of Animal Physiology and Phytopharmacology, University of Dschang, P.O. Box 67, Dschang, Cameroon; 2 Laboratory of Animal Physiology, University of Yaounde 1, P.O. Box 812, Yaounde, Cameroon

**Keywords:** Polycystic ovary syndrome, Female infertility, *Myrianthus arboreus*, Female Wistar rats

## Abstract

**Background:**

*Myrianthus arboreus* P. Beauv. (Cecropiaceae) is a medicinal plant used to treat female infertility. The aqueous extract of *M. arboreus* leaves was found to improve the fertility of healthy female Wistar rats. In the present study, we proposed evaluating the effects of such an extract on an animal model of infertility caused by polycystic ovary syndrome (PCOS), in order to bring scientific evidence to the curative action of this plant against female infertility.

**Methods:**

Following a 21-day administration (gavage) of letrozole (1 mg/kg), animals with PCOS, indicated by overweight and an estrous cycle blocked in the diestrus phase, were co-treated with letrozole (1 mg/kg) and the aqueous extract of *M. arboreus* leaves at doses of 20, 110 and 200 mg/kg. The positive control received clomiphene citrate (1 mg/kg) and metformin (200 mg/kg). The negative control received distilled water. Each group of animals was made up of 10 female rats. Vaginal smear was examined 7 days before and during co-treatments. Co-treatments were orally administered for 30 consecutive days and 5 animals per group were sacrificed thereafter for biochemical and histological analyses. The 5 remaining animals in each group were crossbred with males of proven fertility for 5 consecutive days. The daily examination of vaginal smears allowed evaluating fertility index. Following parturition, gestation rate was calculated.

**Results:**

The aqueous extract of *M. arboreus* leaves reversed letrozole effects by decreasing body weight, abdominal fat accumulation, and serum levels of LH and testosterone (*p* < 0.001). Ovarian dynamic was improved and the number of tertiary, Graafian follicles (*p* < 0.001) and corpus luteum increased while that of cystic (*p* < 0.001) and atretic follicles (*p* < 0.01) decreased. These effects were associated with increased serum levels of estradiol, decreased ovarian oxidative stress, the resumption of the estrous cycle, the hypertrophy of uterine epithelial cells and increased fertility index and gestation rate.

**Conclusions:**

These results justify at least in part, the traditional use of *M. arboreus* against female infertility and suggest that this plant could be a promising alternative treatment to improve symptoms associated with different PCOS phenotypes.

## Background

Polycystic ovary syndrome (PCOS) is one of the most prevalent reproductive endocrinopathies affecting 6–10% of reproductive-age women [1] and 40% of them experience infertility [2]. The latter is known as a failure to conceive after 1 year of appropriately timed unprotected intercourse [3]. In women with PCOS, infertility could result from an impaired ovarian folliculogenesis. Indeed, hyperandrogenism, one of the key features of PCOS, is reported to stimulate an excessive secretion of gonadotropin releasing hormone (GnRH) by hypothalamic neurons and luteinizing hormone (LH) by the pituitary gland [4]. Hyperandrogenism is also reported to induce an excessive release of insulin by pancreatic β-cells [5]. The resulted hyperinsulinemia was found to decrease the hepatic production of insulin-like growth factor (IGF) binding protein-1 and to consequently increase the bioavalability of IGF-1 [6]. Insulin and IGF-1 were found to amplify the effects of LH on granulosa cells, inducing prematurely terminal differentiation [7]. Premature maturation and differentiation of granulosa cells were reported to induce preantral follicular growth arrest, anovulation and cyst formation [7, 8]. All these events could affect fertility in women with PCOS.

Medications like clomiphene citrate, metformin and gonadotropins are commonly used for the treatment of PCOS and PCOS-related infertility. These treatments aimed to increase insulin sensitivity and to improve hyperinsulinemia, hyperandrogenism, ovulatory function and menstrual irregularity [9, 10]. The prescription of metformin, an insulin sensitizer, in polycystic ovarian disease slightly improves ovulation [11], although the conception rate remains disappointing [11, 12]. It is often associated with clomiphene citrate (CC) for a better yield. CC has been used for over 40 years as a triggering agent for first-line ovulation [13, 14]. The literature reports that the concomitant use of CC and metformin significantly increases ovulation and pregnancy rates [11, 12, 15]. Gonadotropins are used when oral medications that trigger ovulation fail to induce ovulation in patients with PCOS [14]. Surgical treatment and in vitro fertilization (IVF) are generally practiced to solve the problem of infertility in patients with PCOS wishing to have children and who did not have a favorable outcome with drug treatments [14, 16]. The use of laparoscopic ovarian drilling (LOD) for instance is considered in women with PCOS resistant to CC. Resistance to CC is evoked in the absence of ovulation or pregnancy following a 6-month treatment [17]. IVF is reserved for women with PCOS who were unable to conceive following treatment with gonadotropins [14].

Although treatments recommended for PCOS effectively relieve symptoms associated with PCOS, they are commonly associated with serious side effects [14, 18, 19]. Moreover, some of them (gonadotropins and surgical interventions) are costly and time-consuming and their use requires intensive monitoring [14], hence the urgency to develop innovative active substances that are better tolerated, more efficient and affordable than the currently applied pharmacological and surgical approaches.

Previous studies reported the efficacy of some plants such as *Aleo barbadensis* [20], *Tephrosia purpurea* [21], *Wistthania somnifera* and *Tribulus terrestris* [22], *Allium fistulosum* [23] and *Phyllanthus muellerianus* [2], in the restoration of insulin sensitivity, hormonal profile and ovarian function in rats with PCOS. These studies suggest that plants with fertilizing and anti-diabetic properties are potential alternatives for the treatment of PCOS. *Myrianthus arboreus* P. Beauv. (Cecropiaceae) for instance, commonly known as “kogom” in Bassa, “angokong” in Bulu and “lilanka” in Dschang, three Cameroonian vernacular languages, is a tree widely distributed in West, Central and East Africa where its young leaves are used for the treatment of various diseases, including diabetes [24–27]. The leaf aqueous extract of *M. arboreus* is used in Cameroon against amenorrhea, female infertility (primary and secondary) and to improve lactation [24, 28, 29]. Kasangana et al. [29] reported that the root bark extracts of *M. arboreus* exhibits anti-diabetic potential in vitro. Additionally, the aqueous extract of *M. arboreus* leaves was found to improve the fertility of healthy female Wistar rats [30]. Although bearing all these properties, *M. arboreus* has not yet been investigated on an animal model of PCOS-related infertility.

In order to test the hypothesis according to which plants with fertilizing and anti-diabetic properties are potential alternatives for the treatment of PCOS, and to bring scientific evidence to the curative action of this plant against female infertility, we investigated the ability of the aqueous extract of *M. arboreus* leaves to relieve PCOS-associated symptoms and to restore the fertility of PCOS animals. In this regard, the effects of the aqueous extract of *M. arboreus* leaves were investigated on body weight, estrous cycle, sex hormones, ovarian histomorphology, oxidative stress-related parameters, uterine growth, fertility index and gestation rate.

## Methods

### Plant material and preparation of the aqueous extract

*Myrianthus arboreus* was collected in October 2018 in Santchou (West region, Cameroon). The plant was authenticated by a botanist, Mr. Victor Nana, at the Cameroon National Herbarium where a voucher specimen has been deposited under the number 34045/HNC.

Following collection, fresh and clean leaves of *M. arboreus* were air-dried (under shade), and ground. One kilogram of the resulting powder was macerated in 10 L of distilled water for 12 h and then filtered with Whatman paper number 4. This first filtrate was stored in a refrigerator unttil use. The same amount of distilled water (10 L) was poured into the residue for an additional maceration of 12 h. Following the filtration of this second macerate, the filtrate obtained was added to the first filtrate. The whole was freeze-dried and a total dry mass of 53.53 g of the aqueous extract was obtained. This extract was kept at 4 °C in an airtight container until use.

Three doses (20, 110 and 200 mg/kg of body weight) of this extract were administered to animals. At these doses, the aqueous extract of *M. arboreus* leaves was reported to stimulate female Wistar rat sexual maturation and to improve the fertility of normocyclic rats, following a 30-day treatment [30]. These doses were extrapolated from the traditional posology against female infertility [31]. The dose of 20 mg/kg was obtained as the therapeutic dose for young adult women [31]. The animal equivalent dose of 200 mg/kg was obtained by multiplying 20 mg/kg by 10 and the dose of 110 mg/kg represents the mean of these two doses (20 and 200 mg/kg) [31].

### Animals

Healthy young female Wistar rats aged 10–12 weeks and weighing 140–160 g prior to the experiment were obtained from the breeding facility of the Research Unit of Animal Physiology and Phytopharmacology, University of Dschang (Cameroon). They were housed in clean plastic cages at room temperature and lit by natural light. All rats had free access to diet (a standard soy-free rat diet in order to eliminate exposure to exogenous estrogenic compounds) and tap water ad libitum.

### Ethical statement

Animal housing and all experiments were conducted in conformity with the European Union on Animal Care (CEECouncil 86/609) guidelines adopted by the Institutional Ethics Committee of the Cameroon Ministry of Scientific Research and Technology Innovation (Reg. no. FWA-IRD 0001954).

### Study design

Sixty female Wistar rats were used to evaluate the effects of the aqueous extract of *M. arboreus* leaves on PCOS-associated symptoms and PCOS-related infertility. PCOS was induced with letrozole following the method described by Ndeingang et al. [2] with slight modifications. Briefly, animals were checked for three consecutive normal estrous cycles by vaginal smear examination [2] and divided into 6 groups of 10 animals each. Group 1 served as the normal control and received the vehicle (distilled water; 1 mL/ 100 g). Animals of groups 2–6 were treated with letrozole (LTZ; 1 mg/kg dissolved in distilled water) for 21 consecutive days for PCOS induction. Seven days before the end of PCOS induction (from day 15 to day 21), vaginal smears were collected from each rat and observed each morning (07:30 a.m. to 08:30 a.m.) to determine their estrous cyclicity, as previously described [2]. At day 22, animals were assigned to the following treatment groups:

- NC (*n* = 10): normal control, healty animals (animals without PCOS) receiving the vehicle (distilled water);
- LTZ (*n* = 10): animals with PCOS receiving letrozole (1 mg/kg) and the vehicle;
- LTZ + CC + MET (*n* = 10): animals with PCOS treated with letrozole (1 mg/kg), clomiphene citrate (1 mg/kg) and metformin (200 mg/kg);
- LTZ + AE20 (n = 10): animals with PCOS treated with letrozole (1 mg/kg) and the aqueous extract of *M. arboreus* leaves at the dose of 20 mg/kg;
- LTZ + AE110 (*n* = 10): animals with PCOS treated with letrozole (1 mg/kg) and the aqueous extract of *M. arboreus* leaves at the dose of 110 mg/kg;
- LTZ + AE200 (n = 10): animals with PCOS treated with letrozole (1 mg/kg) and the aqueous extract of *M. arboreus* leaves at the dose of 200 mg/kg.

Treatments were given orally (every morning from 06:30 a.m. to 07 h30 a.m.) for 30 consecutive days. During this treatment period, the administration of letrozole was not interrupted as the latter is a reversible aromatase inhibitor [4, 22]. Thus, to maintain the cause of PCOS (aromatase inhibition) in experimental animals, letrozole was co-administered with the aqueous extract of *M. arboreus* leaves during the treatment period to determine whether or not the aforementioned extract could improve PCOS symptoms despite the presence of the causal element. The vaginal smear of each rat continued to be examined microscopically from the beginning to the end of treatments. The stages of the estrous cycle were determined by identifying the types of cells present in rat vaginal smears [32]: leucocytes (diestrus); nucleated epithelial cells (proestrus); cornified cells (estrus); and mixed cells (nucleated, cornified, leucocytes) (metestrus). The appearance of these cells depends on the histological changes that the vaginal epithelium undergoes in relation to the hormonal (estradiol) fluctuations occurring at the different stages of the estrous cycle [33, 34]. In addition, our previous studies showed that in animals confined in the same group for several days, the estrous cycles are synchronized and this was evidenced by the appearance of identical histological changes of the vaginal epithelium within the same group [35, 36]. Therefore, results on the estrous cycle in this study, show the most represented phase of the estrous cycle in each group.

At the end of the 30-day treatment, 5 animals in each group were allowed to fast for 12 h and were anesthesized thereafter using an intraperitoneal injection of diazepam (10 mg/kg) and ketamine (50 mg/kg). Following anesthesia, animals were sacrificed by incision of the abdominal artery. Blood was collected from each rat in dry tubes (without an anticoagulant) using a catheter inserted into the aforementioned artery. Blood flowed into dry tubes until death of animals indicated by cessation of breathing and heartbeat. The tubes were kept at room temperature and serum was separated by centrifugation at 3000 rpm for 15 min. The resulting serum was stored at − 20 °C until use. Abdominal fat of each rat were collected and weighed. Ovaries and uteri were collected, cleaned of fat, weighed (only the uterus) and fixed (the whole uterus and the left ovary) in 10% formalin for histological analysis. The right ovary was homogenated in 0.9% NaCl (0.1 g per 1 mL). Tissue homogenates were centrifuged at 3000 rpm for 15 min. The resulting supernatants were stored at − 20 °C until use. Animal body weight was recorded weekly from the beginning to the end of the study.

The remaining female rats in each group (5/group) were crossbred with vigorous males of proven fertility (one male for two females) for 5 consecutive days, duration of an estrous cycle. The vaginal smear was examined daily (07:30 a.m. to 08:30 a.m.) to check the presence of spermatozoa (indicator of gestation) [30]. At the sight of these, female rats were separated from males and this day was considered the first day of gestation [30] and pregnant rats were followed up until parturition. At the end of the study, fertility index and gestational rate were calculated as follows: fertility index = (number of pregnant/number of mated) × 100; gestation rate = (number of females with viable fetuses at birth/total number of gestational females) × 100 [30].

### Biochemical analyses

Serum levels of estradiol, testosterone and luteinizing hormone (LH) were assessed by ELISA tests using reagent kits purchased from Calbiotech (El Cajon, California, USA). Analytical sensitivity for each kit was 5 mIU/ml for LH, 20 ng/ml for estradiol and 0.2 ng/ml for testosterone. The standard curve range was 5–200 mIU/ml for LH, 20–3000 ng/ml for estradiol and 0.2–18 ng/ml for testosterone. The absorbance of calibrators and specimen was determined using an ELISA microplate reader, the Multiskan ascent plate reader, purchased from MTX Lab Systems, Inc. (Brandenton, USA). The concentration was evaluated by means of a calibration curve established from the calibrators supplied with the kits.

Ovarian levels of malondialdehyde (MDA) were determined by the method of Wilbur et al. [37] which is based on the reaction with thiobarbituric acid (TBA) at 90–100 °C. In the TBA test reaction, MDA or MDA-like substances and TBA react with the production of a pink pigment having an absorption maximum at 532 nm. Tissue level of MDA was determined using the following formula: [MDA] = DO/ε.l.m, where [MDA] = concentration of MDA (nM/mg of tissue); DO = absorbance of the sample - absorbance of the reagent blank; ε = molar extinction coefficient (1.56.10^− 4^ nM^− 1^ cm^− 1^); l = path length (1 cm); m = mass of the tissue collected for homogenization (mg).

Ovarian levels of total proteins were assessed using a reagent kit purchased from Randox (London, UK), following the manufacturer’s instructions. This parameter was used to assess tissue levels of antioxidant enzymes (catalase, total peroxidases) as the amount of each of these enzymes in tissue (ovaries) homogenates was assessed relative to total protein content in the aforementioned tissue.

Catalase activity was estimated by the method of Sinha [38] which is based on the decomposition of H_2_O_2_ into water. The concentration of undecomposed H_2_O_2_ was evaluated using a calibration curve established from a standard solution (50 mM H_2_O_2_). Tissue catalase activity was determined as follows: C. A = DO/a.t.p, where: C. A = catalase activity (mole of H_2_O_2_/min/g of total proteins); DO = absorbance of the sample - absorbance of the reagent blank; a = slope of the calibration curve; t = reaction time (1 min); p = ovarian total protein level (g).

Ovarian levels of total peroxidases were determined by the method of Habbu et al. [39] and the method description partly reproduces their wording. Briefly, ovarian homogenate (0.5 ml) was taken, and to this were added 1 ml KI solution (10 mM) and 1 ml sodium acetate (40 mM). The absorbance of potassium iodide was read at 353 nm, which indicates the amount of peroxidase. Then 20 μl of H_2_O_2_ (15 mM) was added, and the change in the absorbance in 5 min was recorded. Units of peroxidase activity were expressed as the amount of enzyme required to change the optical density by 1 unit per min. The specific activity expressed in terms of units per g of proteins was deduced by the law of Beer-Lambert [40] as follows: C = DO/ε.l.p, where C = concentration of ovarian total peroxidases (mM/g of total proteins); DO = optical density; ε = molar extinction coefficient (11.3 M^− 1^ cm^− 1^); l = path length (1 cm); p = ovarian total protein level (g).

### Histological analysis

Histological analyses of the ovary and the uterus were assessed from 5 μm sections of paraffin embedded tissues following hematoxylin-eosin staining. Histomorphological changes were assessed on microphotographs using a Scientico STM-50 microscope equipped with a Celestron MA411101 camera connected to a computer where the image was transferred and analyzed with the Image J1.3 software.

### Statistical analysis

Data were analyzed using the GraphPad Prism 5.03 software and are presented as mean ± standard error of the mean (S.E.M.), except data of the estrous cycle which rather show the most represented phase of the estrous cycle in each group, as reported by Belani et al. [32]. Statistical significance and the difference among groups were evaluated by one-way analysis of the variance (ANOVA) followed by Tukey test for multiple comparisons. Differences were considered significant at *p* < 0.05.

## Results

### Body weight and relative abdominal fat weight

Table 1 shows that animal body weight before PCOS induction was similar in all groups. Following a 21-day administration of letrozole, animal body weight increased by at least 12% (*p* < 0.001) as compared with the normal control group. The combined administration of clomiphene citrate and metformin reversed the effect of letrozole as it decreased animal body weight by 26% (*p* < 0.001), as compared with the LTZ group. The aqueous extract of *M. arboreus* leaves induced a similar effect at the dose of 110 mg/kg, as it decreased animal body weight by 17% (*p* < 0.001) as compared with the LTZ group.

**Table 1:** Effects of the leaf aqueous extract of *M. arboreus* on body and abdominal fat weights

Groups	Body weight before letrozole administration (g)	Body weight following a 21-day administration of letrozole (g)	Body weight following a 30-day treatment (g)	Abdominal fat weight (g/100 g of BW)
**NC**	161.60 ± 2.38	164.75 ± 0.69	176.60 ± 3.63	2.52 ± 0.32
**LTZ**	159.89 ± 2.29	186.50 ± 1.98^***^	195.50 ± 9.93^*^	3.55 ± 0.16
**LTZ + CC + MET**	158.71 ± 2.74	186.50 ± 1.98^***^	144.50 ± 20.36^***, ###^	0.61 ± 0.09^***, ###^
**LTZ + AE 20**	162.38 ± 2.78	183.40 ± 2.30^***^	190.33 ± 12.07	2.43 ± 0.45
**LTZ + AE 110**	157.90 ± 3.31	186.50 ± 1.98^***^	162.44 ± 17.61^###^	2.37 ± 0.23^#^
**LTZ + AE 200**	157.90 ± 3.31	184.60 ± 1.96^***^	187.12 ± 12.03	3.17 ± 0.18

*NC* normal control; *LTZ* letrozole; *CC* clomiphene citrate; *MET* metformine; *AE* leaf aqueous extract of *M. arboreus*; *BW* body weight. Results are presented as mean ± S.E.M., *n* = 5. * *p* < 0.05 and *** *p* < 0.001 vs NC; # *p* < 0.05 and ### *p* < 0.001 vs LTZ

The relative weight of the abdominal fat increased by 41% in the LTZ group as compared with the normal control group (Table 1). The combined administration of clomiphene citrate and metformin decreased this parameter by 83% (*p* < 0.001) as compared with the LTZ group. The aqueous extract of *M. arboreus* leaves induced a similar effect at tested doses.

### Estrous cycle

**Fig. 1:**
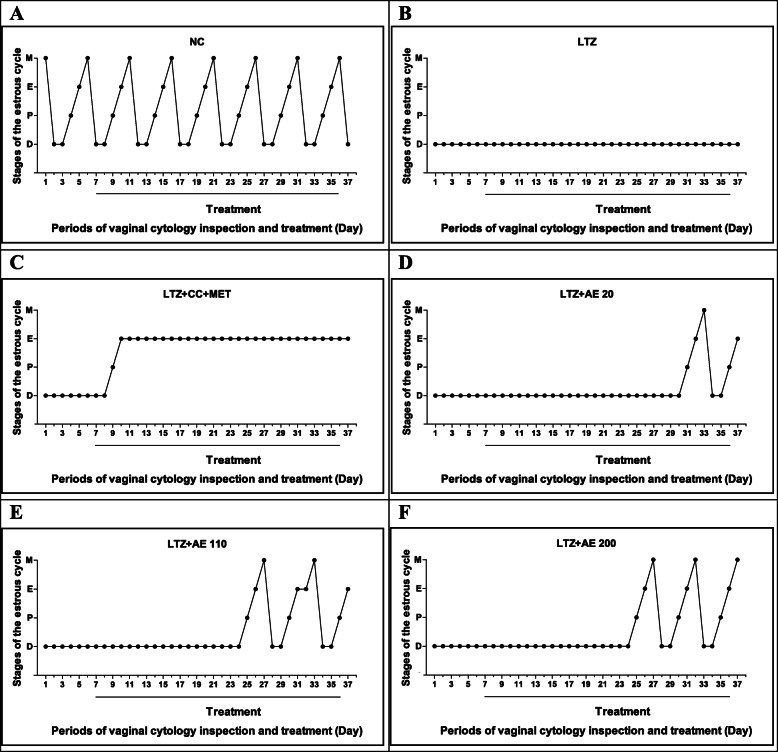
Effects of the leaf aqueous extract of *M. arboreus* on the estrous cycle. NC: normal control; LTZ: letrozole; CC: clomiphene citrate; MET: metformine; AE: leaf aqueous extract of *M. arboreus*. Data show the most represented phase of the estrous cycle in each group, *n* = 5. P = proestrous stage-nucleated epithelial cells, E = estrous stage-cornified cells, M = metestrus-nucleated, cornified and leucocytes, and D = diestrous stage-leucocytes

Figure 1a shows that the estrous cycle of animals in the normal control group lasted on average 5 days and consisted of the following successive estrous phases: proestrus, estrus, metestrus and two phases of diestrus. In the LTZ group, the estrous cycle was blocked in the diestrus phase (Fig. 1b). The cyclic appearance of the different phases of the estrous cycle, starting with the proestrus phase, resumed in animals receiving both clomiphene citrate and metformin after 2 days of administration (Fig. 1c). Following treatment with the aqueous extract of *M. arboreus* leaves, proestrus appeared after 24 days of administration at the dose of 20 mg/kg (Fig. 1d) and after 18 days of administration at doses of 110 and 200 mg/kg, respectively (Fig. 1e and f).

### Serum levels of testosterone, LH and estradiol

**Fig. 2:**
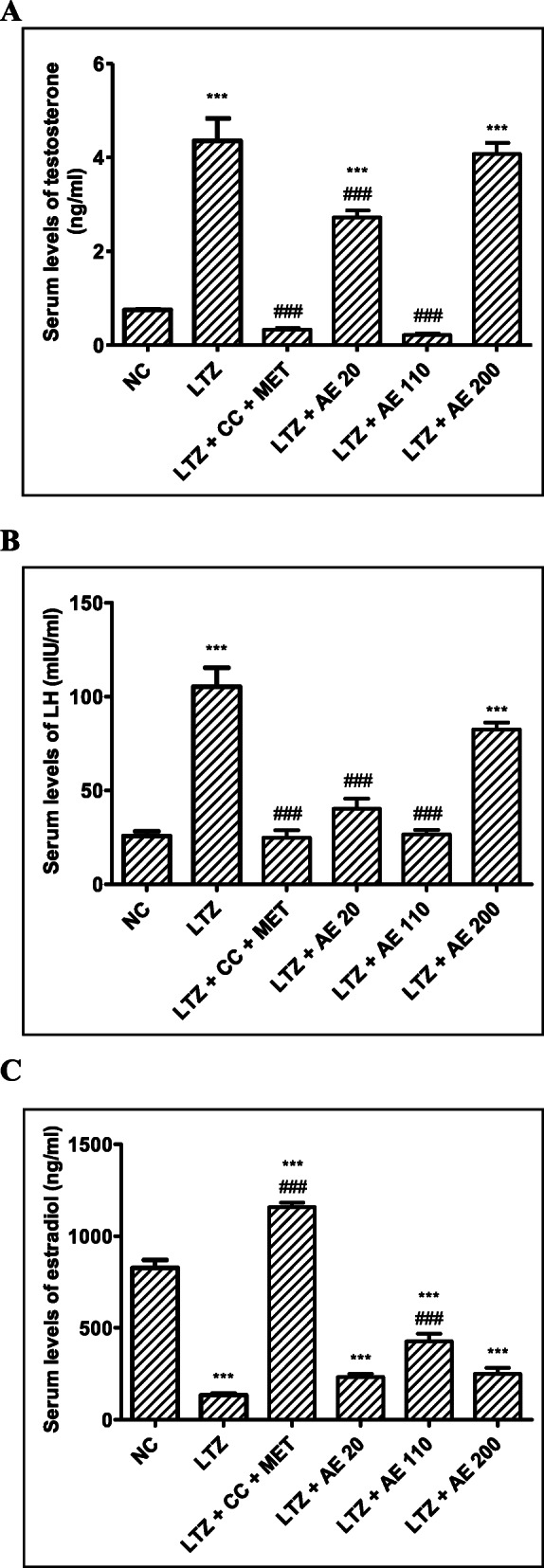
Serum levels of testosterone (**a**), LH (**b**) and estradiol (**c**) following treatments. NC: normal control; LTZ: letrozole; CC: clomiphene citrate; MET: metformine; AE: leaf aqueous extract of *M. arboreus*. Results are presented as mean S.E.M., *n* = 5. *** *p* < 0.001 vs NC; ### *p* < 0.001 vs LTZ

Serum levels of testosterone increased by 481% (*p* < 0.001) in the LTZ group as compared with the normal control group (Fig. 2a). The combined administration of clomiphene citrate and metformin decreased this parameter by 92% (*p* < 0.001) as compared with the LTZ group. The aqueous extract of *M. arboreus* leaves induced a similar effect at doses of 20 mg/kg (37% induction; *p* < 0.001) and 110 mg/kg (95% induction; *p* < 0.001).

Figure 2b shows that serum levels of LH increased by 310% (*p* < 0.001) in the LTZ group as compared with the normal control group. The combined administration of clomiphene citrate and metformin decreased this parameter by 77% (*p* < 0.001) in comparison with the LTZ group. The aqueous extract of *M. arboreus* leaves induced a similar effect at tested doses.

Figure 2c shows that serum levels of estradiol decreased by 84% (*p* < 0.001) in the LTZ group as compared with the normal control group. The combined administration of clomiphene citrate and metformin increased this parameter by 764% (*p* < 0.001) as compared with the LTZ group. The aqueous extract of *M. arboreus* leaves also induced a similar effect as it increased serum levels of estradiol by 74, 219 and 87% at doses of 20, 110 and 200 mg/kg, respectively.

### Histological score of the ovaries

The histological score of the ovaries presented in Table 2 shows that the number of primary follicles decreased by 53% (*p* < 0.05) in the LTZ group as compared with the normal control group. The combined administration of clomiphene citrate and metformin reversed this effect as it increased the number of primary follicles by 233% (*p* < 0.001) in comparison with the LTZ group. A similar effect was observed with the aqueous extract of *M. arboreus* leaves which increased the number of primary follicles by 300, 433 and 500% (*p* < 0.001) at doses of 20, 110 and 200 mg/kg, respectively, as compared with the LTZ control.

**Table 2:** Effects of the leaf aqueous extract of *M. arboreus* on ovarian follicle growth and maturation

Groups	Primary follicles	Secondary follicles	Tertiary follicles	Graafian follicles	Corpora lutea	Cystic follicles	Atretic follicles
**NC**	3.20 ± 0.49	2.80 ± 0.20	2.20 ± 0.37	2.40 ± 0.24	12.00 ± 1.26	0.00 ± 0.00	5.60 ± 0.51
**LTZ**	1.50 ± 0.22^*^	0.80 ± 0.37	1.40 ± 0.24	1.60 ± 0.24	4.75 ± 0.19^***^	13.25 ± 0.79^***^	10.60 ± 0.60^***^
**LTZ + CC + MET**	5.00 ± 0.32^*, ###^	4.75 ± 0.37^#^	6.00 ± 0.45^***, ###^	3.80 ± 0.58^##^	10.00 ± 1.14^###^	2.33 ± 0.18^**, ###^	7.00 ± 0.45 ^###^
**LTZ + AE 20**	6.00 ± 0.32^***, ###^	5.00 ± 0.32^#^	3.33 ± 0.18^#^	5.00 ± 0.32^***, ###^	9.50 ± 0.50^##^	3.67 ± 0.36^***, ###^	7.20 ± 0.37^##^
**LTZ + AE 110**	8.00 ± 0.32^***, ###^	8.80 ± 1.07^***, ###^	4.00 ± 0.32^*, ###^	5.25 ± 0.19^***, ###^	6.20 ± 0.58^***^	4.00 ± 0.32^***, ###^	6.60 ± 0.81^###^
**LTZ + AE 200**	9.00 ± 0.55^***, ###^	9.20 ± 1.59^***, ###^	3.40 ± 0.51^##^	6.00 ± 0.32^***, ###^	7.33 ± 0.36^**^	4.00 ± 0.32^***, ###^	7.60 ± 0.25^##^

*NC* normal control; *LTZ* letrozole; *CC* clomiphene citrate; *MET* metformine; *AE* leaf aqueous extract of *M. arboreus*. Results are presented as mean S.E.M., *n* = 5. ^*^ *p* < 0.05, ^**^ *p* < 0.01 and ^***^ *p* < 0.001 vs NC. ^#^ *p* < 0.05, ^##^ *p* < 0.01 and ^###^ *p* < 0.001 vs LTZ

The number of secondary follicles decreased by 71% in the LTZ group as compared with the normal control group. The combined administration of clomiphene citrate and metformin increased this parameter by 494% (*p* < 0.05) in comparison with the LTZ group. A similar effect was observed with the aqueous extract of *M. arboreus* leaves at tested doses.

The number of tertiary follicles decreased by 36% in the LTZ group as compared with the normal control group. Following the combined administration of clomiphene citrate and metformine, the number of tertiary follicles increased by 329% (*p* < 0.001) in comparison with the LTZ group. A similar observation was made with the aqueous extract of *M. arboreus* leaves at tested doses.

The number of Graafian follicles decreased by 33% in the LTZ group as compared with the normal control group. Following the combined administration of clomiphene citrate and metformin, the number of Graafian follicles increased by 138% (*p* < 0.01) in comparison with the LTZ group. A similar observation was made with the aqueous extract of *M. arboreus* leaves at tested doses.

The number of corpora lutea decreased by 60% (*p* < 0.001) in the LTZ group as compared with the normal control group. The combined administration of clomiphene citrate and metformin increased it by 110% (*p* < 0.001) in comparison with the LTZ group. A similar observation was made with the aqueous extract of *M. arboreus* leaves at tested doses.

The number of atretic follicles increased by 89% (*p* < 0.001) in the LTZ group as compared to the normal control group. The combined administration of clomiphene citrate and metformin decreased it by 34% (*p* < 0.001) in comparison with the LTZ group. The aqueous extract of *M. arboreus* leaves induced a similar effect at tested doses.

In the ovaries of normal control animals, no cystic follicles were identified. However, in the ovaries of animals in the LTZ group, the number of cystic follicles was on average 13.25 ± 0.80 (*p* < 0.001). This number decreased by 82% (*p* < 0.001) following the co-treatment with clomiphene citrate and metformin, in comparison with the LTZ group. The aqueous extract of *M. arboreus* leaves induced a similar effect at tested doses.

**Fig. 3:**
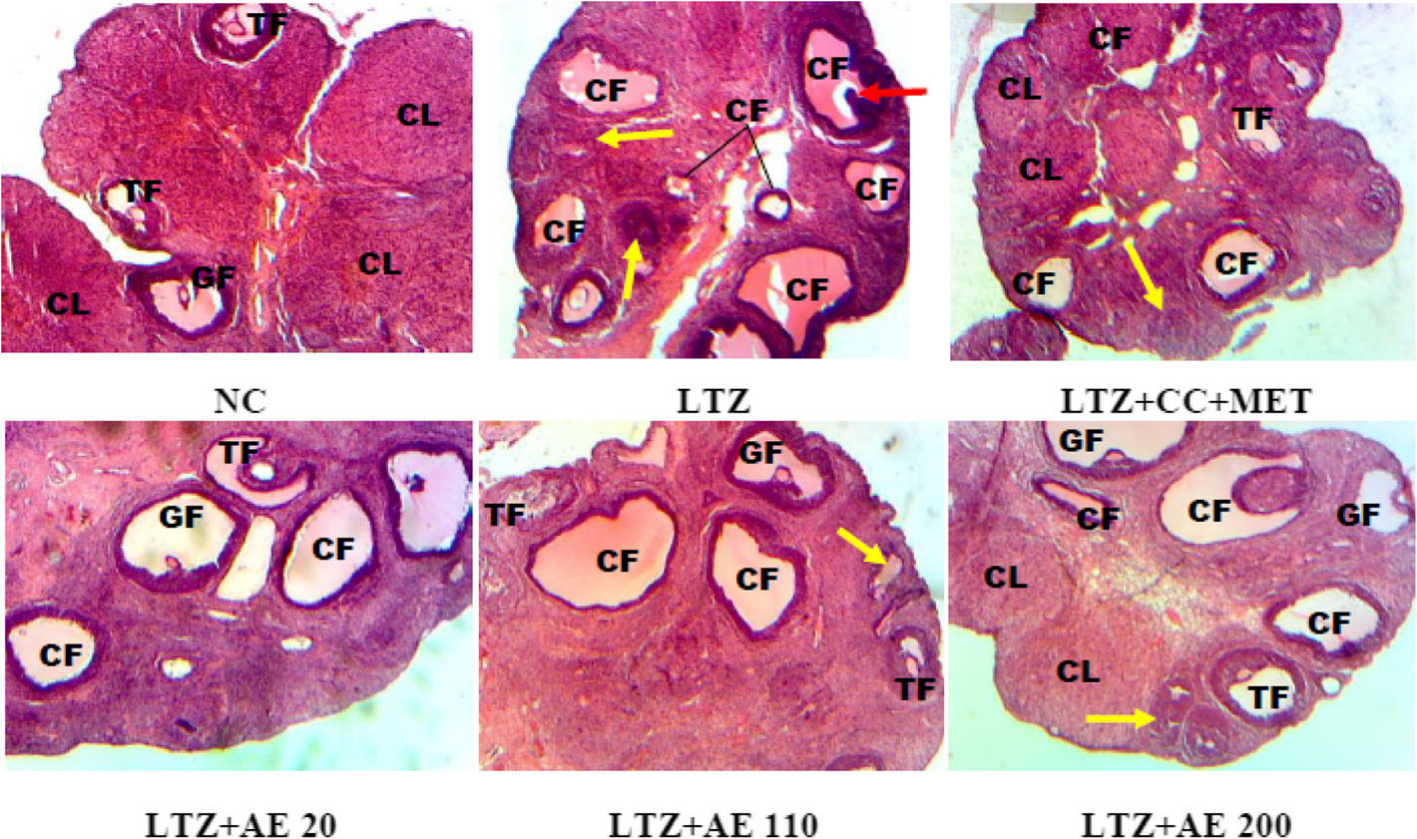
Photomicrographs (X 200, hematoxylin and eosin staining) of the ovaries of experimental animals. NC: normal control; LTZ: letrozole; CC: clomiphene citrate; MET: metformine; AE: leaf aqueous extract of *M. arboreus*. TF: tertiary follicles; GF: Graafian follicles; CL: corpus luteum; CF: cystic follicles; red arrow: degenerating oocyte; yellow arrow: atretic follicles

Figure 3 shows photomicrographs of the ovaries of experimental animals where the following follicles are identified: tertiary follicles, Graafian follicles, corpus luteum, cystic and atretic follicles. Ovarian sections of the normal control group exhibited normal ovarian morphology with mature follicles (tertiary and Graafian follicles) and corpora lutea which is an indicator of ovulation. In PCOS animals (in the LTZ group), the microarchitecture of the ovaries was dominated by the presence of cystic and atretic follicles. Following treatments, ovarian sections exhibited mature follicles, corpora lutea and few cystic and atretic follicles.

### Ovarian levels of malondialdehyde, catalase and total peroxidases

Ovarian levels of malondialdehyde (MDA) increased by 16% in the LTZ group as compared with the normal control group (Table 3). The combined administration of clomiphene citrate and metformin decreased this parameter by 33% (*p* < 0.01) in comparison with the LTZ group. The aqueous extract of *M. arboreus* leaves induced a similar effect at doses of 20 mg/kg (27% induction, *p* < 0.05) and 110 mg/kg (46% induction, *p* < 0.001), respectively.

**Table 3:** Ovarian levels of malondialdehyde, catalase and total peroxydases, following treatments

Groups	Malondialdehyde (μM)	Catalase (μmole/g of total proteins)	Total peroxidases (mM/g of total proteins)
**NC**	1.60 ± 0.14	2.09 ± 0.11	11.13 ± 1.32
**LTZ**	1.86 ± 0.11	1.57 ± 0.03^***^	6.64 ± 0.37^**^
**LTZ + CC + MET**	1.25 ± 0.09^##^	1.47 ± 0.11^***^	7.53 ± 0.38^*^
**LTZ + AE 20**	1.36 ± 0.032^#^	1.96 ± 0.07^#^	7.84 ± 0.06
**LTZ + AE 110**	1.01 ± 0.10^**, ###^	1.79 ± 0.05	8.27 ± 0.71
**LTZ + AE 200**	1.73 ± 0.08	1.79 ± 0.05	10.17 ± 1.12^#^

*NC* normal control; *LTZ* letrozole; *CC* clomiphene citrate; *MET* metformine; *AE* leaf aqueous extract of *M. arboreus*. Results are presented as mean S.E.M., *n* = 5. ^*^ *p* < 0.05, ^**^ *p* < 0.01 and ^***^ *p* < 0.001 vs NC. ^#^ *p* < 0.05, ^##^ *p* < 0.01 and ^###^ *p* < 0.001 vs LTZ

The ovarian level of catalase decreased by 25% (*p* < 0.001) in the LTZ group as compared with the normal control group (Table 3). The combined administration of clomiphene citrate and metformin did not alter ovarian level of catalase which remained similar to that of the LTZ group. The aqueous extract of *M. arboreus* leaves, in contrast, increased this parameter by 25% (*p* < 0.05) at the dose of 20 mg/kg and 14% at doses of 110 and 200 mg/kg, in comparison with the LTZ group.

Table 3 also shows that the ovarian level of total peroxidases decreased by 40% (*p* < 0.01) in the LTZ group as compared with the normal control group. The combined administration of clomiphene citrate and metformin increased this parameter by 13% in comparison with the LTZ group. A similar effect was observed with the aqueous extract of *M. arboreus* leaves which increased ovarian level of total peroxidases by 18, 25 and 53% at doses of 20, 110 and 200 mg/kg, respectively, in comparison with the LTZ group.

### Relative uterine weight and uterine epithelial height

**Fig. 4:**
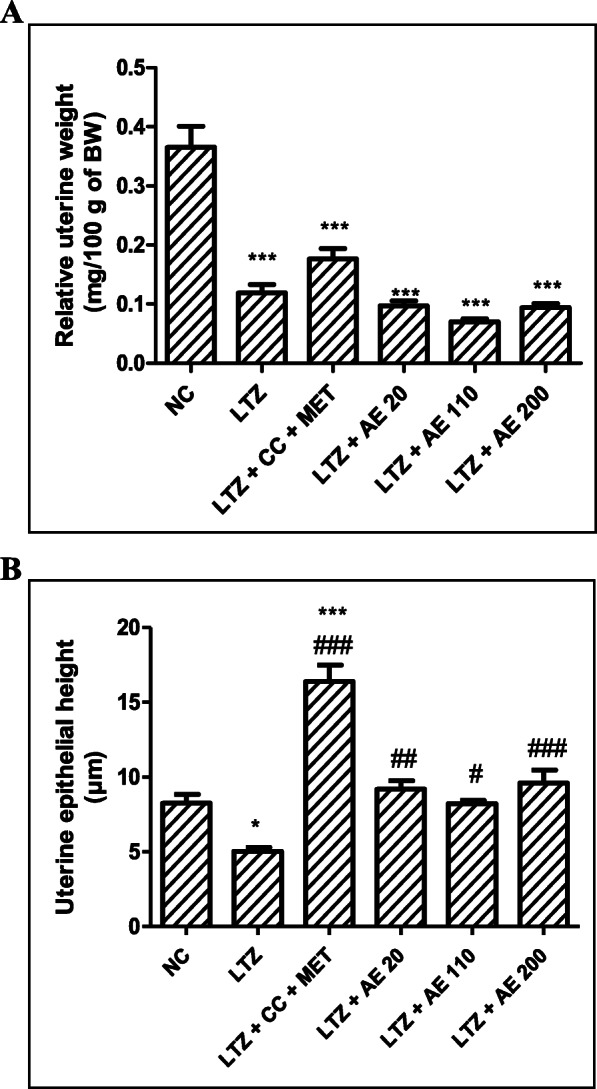
Relative uterine weight (**a**) and uterine epithelial height (**b**) following treatments. NC: normal control; LTZ: letrozole; CC: clomiphene citrate; MET: metformine; AE: leaf aqueous extract of *M. arboreus*. Results are presented as mean S.E.M., *n* = 5. ^*^ *p* < 0.05 and ^***^ *p* < 0.001 vs NC; ^#^ *p* < 0.05, ^##^ *p* < 0.01 and ^###^ *p* < 0.001 vs LTZ

Letrozole (LTZ) decreased the relative uterine weight by 67% (*p* < 0.001) as compared with the normal control group (Fig. 4a). The combined administration of clomiphene citrate and metformin increased this parameter by 48% as compared with the LTZ group. In contrast, following treatment with the aqueous extract of *M. arboreus* leaves, the relative uterine weight further decreased: 18% induction at 20 mg/kg, 41% induction at 110 mg/kg and 21% induction at 200 mg/kg.

The uterine epithelial height of animals in the LTZ group decreased by 39% (*p* < 0.05) as compared with the normal control group (Fig. 4b). Co-treatment with clomiphene citrate and metformin increased this parameter by 225% (*p* < 0.001) as compared with the LTZ group. The aqueous extract of *M. arboreus* leaves induced a similar effect as it increased uterine epithelial height by 82 (*p* < 0.01), 63 (*p* < 0.05) and 90% (*p* < 0.001) at 20, 110 and 200 mg/kg, respectively, in comparison with the LTZ group.

**Fig. 5:**
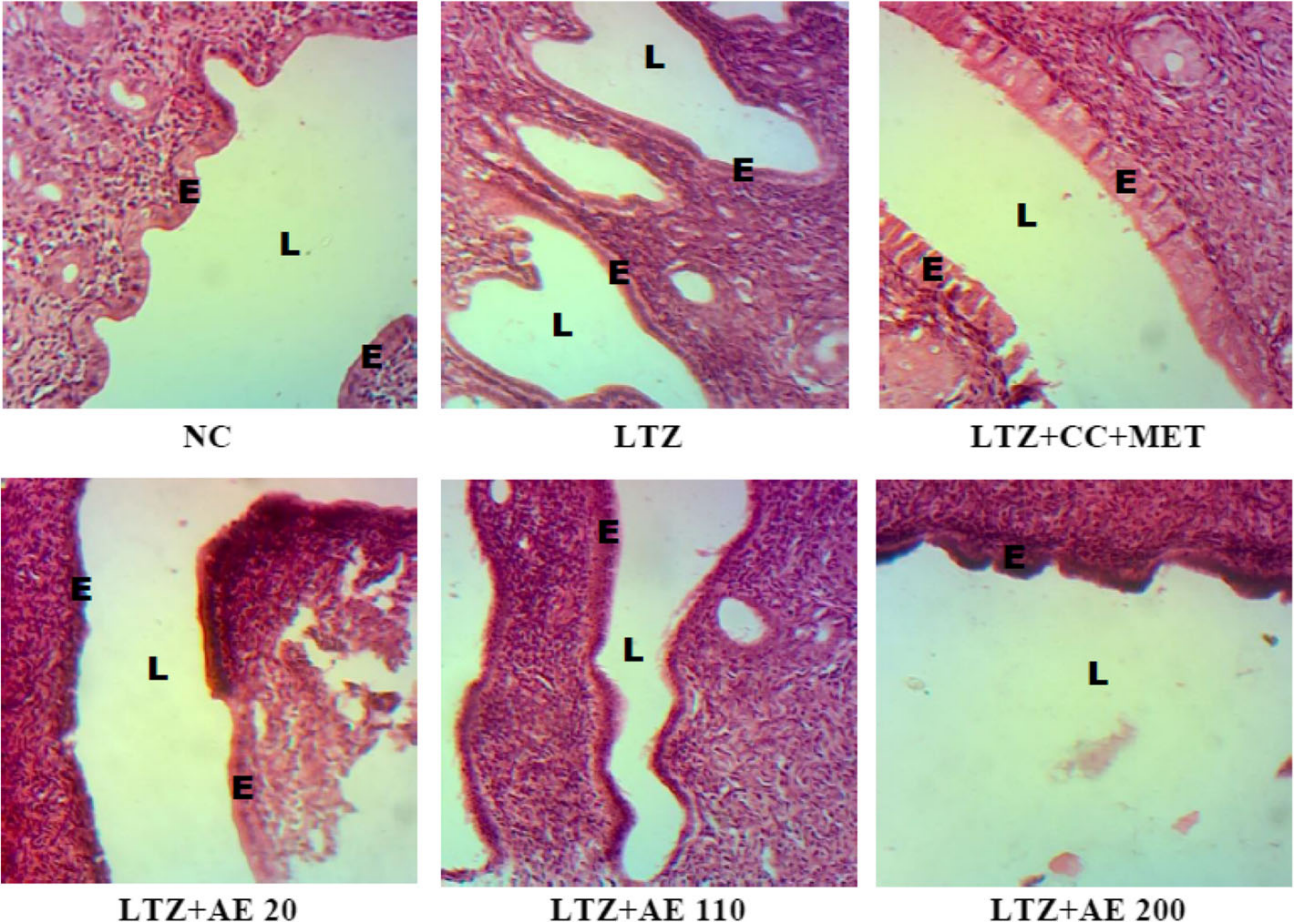
Photomicrographs (X 100, hematoxylin and eosin staining) of the uterus of experimental animals. NC: normal control; LTZ: letrozole; CC: clomiphene citrate; MET: metformine; AE: leaf aqueous extract of *M. arboreus*. BW: body weight; E: epithelium; L: lumen

Figure 5 shows that the uterus of animals in the normal control group was lined by a tall cuboidal epithelium. In animals of the LTZ group, the uterus was lined by a low cuboidal epithelium. Co-treatment with clomiphene citrate and metformin changed uterine epithelial cell morphology that became cylindrical. Treatment with the aqueous extract of *M. arboreus* leaves also induced the hypertrophy of uterine epithelial cells at tested doses.

### Fertility index and gestation rate

Fertility index went from 66.67% in the normal control group to 0% in the LTZ group (Table 4). Co-treatment with clomiphene citrate and metformin, as well as the aqueous extract of *M. arboreus* leaves, increased this parameter by at least 66.67%.

**Table 4:** Effects of the leaf aqueous extract of *M. arboreus* on fertility index and gestation rate

Groups	Fertility index (%)	Gestation rate (%)
**NC**	66.67	100
**LTZ**	0	0
**LTZ+ CC+ MET**	75	100
**LTZ + AE 20**	66.67	100
**LTZ + AE 110**	66.67	100
**LTZ + AE 200**	75	100

*NC* normal control; *LTZ* letrozole; *CC* clomiphene citrate; *MET* metformine; *AE* leaf aqueous extract of *M. arboreus*

Gestation rate went from 100% in the normal control group to 0% in the LTZ group (Table 4). Co-treatment with clomiphene citrate and metformin, as well as the aqueous extract of *M. arboreus* leaves, increased gestation rate which went from 0% in the LTZ group to 100% following treatment.

## Discussion

The present study aimed to investigate the ability of the aqueous extract of *M. arboreus* leaves to relieve PCOS-related conditions including infertility, in rats. PCOS was induced with letrozole following the method described by Ndeingang et al. [2]. Since letrozole is a reversible aromatase inhibitor [4, 22], its administration was not interrupted during treatments which lasted 30 days. In agreement with previous reports [2, 20, 22, 23], results showed that letrozole induced overweight, blocked the estrous cycle in the diestrus phase, increased serum levels of testosterone and LH, promoted ovarian cyst formation and induced infertility. By inhibiting aromatase activity, letrozole induces an accumulation of androgens in the blood stream leading to hyperandrogenism [41] with the resulting blockage of the estrous cycle in the diestrus phase, since vaginal smears undergo cytological changes only according to the cyclical variations of serum estradiol levels [34]. Excess androgen stimulates an excessive pituitary secretion of LH by promoting an overproduction of GnRH by the hypothalamic neurons [4]. Indeed, hyperandrogenism was found to induce an excessive release of insulin by pancreatic β-cells [5]. High quantities of insulin were reported to increase glutamate levels in the brain tissue of animals with letrozole-induced PCOS, and elevated amounts of this excitatory neurotransmitter are known to induce an overstimulation of GnRH and LH release [4]. Hyperinsulinemia was also suggested to induce weight gain as insulin was found to inhibit the activity of adenosine monophosphate-activated protein kinase (AMPK) [42], resulting in the enhancement of acetyl CoA carboxylase (ACC) activity, which promotes the biosynthesis of fatty acids [43, 44]. The resulting fat accumulation might account for the observed weight gain following letrozole administration. In addition, insulin increases the bioavailability of insulin-like growth factor 1 (IGF-1) and both insulin and IGF-1 were reported to amplify the effects of LH on granulosa cells, leading to their premature differentiation which results in follicular growth arrest, anovulation and cyst formation [7, 8]. All these events would have contributed to reduce fertility index and gestation rate in PCOS rats.

Following treatments, the aqueous extract of *M. arboreus* leaves decreased animal body weight and reduced fat accumulation. These results suggest that *M. arboreus* would have increased the activity of AMPK in peripheral tissues and therefore, reduced the activity of ACC. Low activity of ACC was associated with reduced lipogenesis and weight loss [43, 44]. *M. arboreus* also induced the resumption of the estrous cycle after it was arrested in the diestrus phase following letrozole administration. This result was associated with decreased levels of LH and testosterone, increased levels of estradiol, and a restored ovarian dynamic (improved ovarian follicle growth and maturation, reduction of follicle atresia, and development and maintenance of the corpora lutea). These results indicate that *M. arboreus* has reduced the inhibitory effect of letrozole on aromatase activity leading to decreased levels of testosterone and LH, and increased levels of estradiol. This estradiol would have stimulated uterine epithelial cells growth. Such an uterotrophic effect is known to be an estrogen receptor alpha mediated event [45, 46]. Moreover, the improvement of ovarian dynamic suggests that *M. arboreus* would have lowered androgen stimulation of insulin secretion by pancreatic β-cells as it reduced circulating free androgen. Since hyperinsulinemia induced by hyperandrogenism was associated with premature maturation and luteinization of granulosa cells, follicular growth arrest, anovulation and cyst formation [7, 8], results suggest that, by reducing testosterone level and probably insulin production by pancreatic β-cells, *M. arboreus* would have consequently reduced the hyperinsulinemic signal impairing ovarian dynamic, and would have restored it.

The restoration of ovarian dynamic was characterized by the appearance of increased number of Graafian follicles (indicating follicular development and maturation), corpora lutea (the hallmark of ovulation), and reduced number of atretic follicles. Follicular atresia observed in PCOS rats was attributed to increased apoptosis of granulosa cells as a result of low estradiol levels [47]. Oxidative stress was reported to initiate cell apoptosis [48], and substances with antioxidant properties were found reducing oxidative stress [47, 49, 50] and follicular atresia [47, 50] in PCOS rats. Additionally, estradiol was reported to be a survival factor for the maintenance of granulosa cells [47]. Therefore, low levels of estradiol in PCOS animals would have promoted oxidative stress-mediated granulosa cell apoptosis and the resulting follicular atresia. By increasing the production of estradiol, *M. arboreus* would have reduced oxidative stress in the ovaries of treated animals as evidenced by low levels of malondialdehyde (MDA, a lipid peroxidation byproduct and a marker of oxidative stress) and increased levels of antioxidant enzymes (catalase and total peroxidases), thus stimulating the resumption of follicular development and maturation, as shown by the increased number of Graafian follicles. This ability of *M. arboreus* to reduce oxidative stress in the ovaries of PCOS animals may be also attributed to its reported antioxidative properties [29, 51, 52].

The effects induced by the aqueous extract of *M. arboreus* leaves on serum levels of hormones and ovarian dynamic would have contributed to the resumption of the estrous cycle, the hypertrophy of uterine epithelial cells and finally the restoration of PCOS rat fertility. Indeed, results showed that fertility index and gestation rate increased in animals treated with the aqueous extract of *M. arboreus* leaves in comparison with those receiving letrozole only, in which both parameters were null. Following treatment with the aqueous extract of *M. arboreus* leaves, more than 66% of female rats were pregnant and 100% of gestational female rats had viable and healthy fetuses at birth. These results corrorate the observations previously made by Awounfack et al. [30] in healthy animals and further support ethnobotanical data reporting the efficacy of *M. arboreus* in the treatment of female infertility [24, 28–30].

## Conclusions

The present study showed that the aqueous extract of *M. arboreus* leaves improved PCOS-associated conditions as it reduced weight gain, restored estrous cycle, decreased abdominal fat accumulation, and serum levels of testosterone and LH. *M. arboreus* also increased serum levels of estradiol which would have contributed to induce uterine epithelial cell hypertrophy, and to reduce ovarian oxidative stress and follicular atresia. All these events would have contributed to restore ovarian dynamic and to increase fertility index and gestation rate in treated animals. These results support at least in part, the traditional use of *M. arboreus* against female infertility and suggest that this plant could be a promising alternative treatment to improve symptoms associated with different PCOS phenotypes.

## Data Availability

All the data used to support the findings of this study are available from the corresponding author upon reasonable request.

## Abbreviations

PCOS: Polycystic ovarian syndrome
GnRH: Gonadotropin releasing hormone
LH: Luteinizing hormone
IGF-1: Insulin-like growth factor 1
IVF: In vitro fertilization
LOD: Laparoscopic ovarian drilling
SEM: Standard error of the mean
NC: Normal control
LTZ: Letrozole
CC: Clomiphene citrate
MET: Metformin
EA: Leaf aqueous extract of *M. arboreus*
E: Uterine epithelium
L: Uterine lumen
MDA: Malondialdehyde
SOD: Superoxide dismutase
